# Gene-based SSR markers for common bean (*Phaseolus vulgaris *L.) derived from root and leaf tissue ESTs: an integration of the BMc series

**DOI:** 10.1186/1471-2229-11-50

**Published:** 2011-03-22

**Authors:** Matthew W Blair, Natalia Hurtado, Carolina M Chavarro, Monica C Muñoz-Torres, Martha C Giraldo, Fabio Pedraza, Jeff Tomkins, Rod Wing

**Affiliations:** 1 CIAT - International Center for Tropical Agriculture, Biotechnology Unit and Bean Project, AA6713, Cali, Valle, Colombia; 2 Clemson University Genomics Institute, Clemson, South Carolina, USA; 3 Department of Biology, Georgetown University, Washington DC, USA; 4 Department of Plant Pathology, Kansas State University, Manhattan, Kansas, USA; 5 Sun Seeds, Fargo ND, USA; 6 Arizona Genomics Institute, Tuscon, Arizona, USA

## Abstract

**Background:**

Sequencing of cDNA libraries for the development of expressed sequence tags (ESTs) as well as for the discovery of simple sequence repeats (SSRs) has been a common method of developing microsatellites or SSR-based markers. In this research, our objective was to further sequence and develop common bean microsatellites from leaf and root cDNA libraries derived from the Andean gene pool accession G19833 and the Mesoamerican gene pool accession DOR364, mapping parents of a commonly used reference map. The root libraries were made from high and low phosphorus treated plants.

**Results:**

A total of 3,123 EST sequences from leaf and root cDNA libraries were screened and used for direct simple sequence repeat discovery. From these EST sequences we found 184 microsatellites; the majority containing tri-nucleotide motifs, many of which were GC rich (ACC, AGC and AGG in particular). Di-nucleotide motif microsatellites were about half as common as the tri-nucleotide motif microsatellites but most of these were AG_n _microsatellites with a moderate number of AT_n _microsatellites in root ESTs followed by few AC_n _and no GC_n _microsatellites. Out of the 184 new SSR loci, 120 new microsatellite markers were developed in the BMc (Bean Microsatellites from cDNAs) series and these were evaluated for their capacity to distinguish bean diversity in a germplasm panel of 18 genotypes. We developed a database with images of the microsatellites and their polymorphism information content (PIC), which averaged 0.310 for polymorphic markers.

**Conclusions:**

The present study produced information about microsatellite frequency in root and leaf tissues of two important genotypes for common bean genomics: namely G19833, the Andean genotype selected for whole genome shotgun sequencing from race Peru, and DOR364 a race Mesoamerica subgroup 2 genotype that is a small-red seeded, released variety in Central America. Both race Peru and Mesoamerica subgroup 2 (small red beans) have been understudied in comparison to race Nueva Granada and Mesoamerica subgroup 1 (black beans) both with regards to gene expression and as sources of markers. However, we found few differences between SSR type and frequency between the G19833 leaf and DOR364 root tissue-derived ESTs. Overall, our work adds to the analysis of microsatellite frequency evaluation for common bean and provides a new set of 120 BMc markers which combined with the 248 previously developed BMc markers brings the total in this series to 368 markers. Once we include BMd markers, which are derived from GenBank sequences, the current total of gene-based markers from our laboratory surpasses 500 markers. These markers are basic for studies of the transcriptome of common bean and can form anchor points for genetic mapping studies in the future.

## Background

Genic microsatellites are those microsatellites based on simple sequence repeats (SSRs) found within, or closely associated with, gene sequences from a given genome [1]. These SSRs tend to be more conserved and of different motifs than SSRs located in other non-gene containing regions of the genome, which are often referred to as genomic microsatellites simply to distinguish them from genic microsatellites [2]; although both gene and non-gene derived microsatellites are obviously part of the overall genome. Simple sequence repeats are defined as small stretches of repeated DNA, usually of two to six nucleotides, tandemly repeated and located in a given pattern between segments of non-repeated DNA [3]. In practice, remnant repeats can be found on either side of a stretch of SSR and in some occasions different motifs are combined together or either motif is interrupted [4]. This differentiates microsatellites into compound or simple microsatellites in the first case, and perfect and imperfect microsatellites in the latter case [5].

Common bean, *Phaseolus vulgaris *L., is an important food legume, basic to the diet of the poor in tropical regions of the world, and a major source of income for small farmers there. Genic microsatellites have been limited in number for this crop. This is perhaps due to two main reasons: 1) a lack of funding has precluded large scale expressed sequence tag (EST) sequencing or even the sufficient construction of many cDNA libraries for the crop and 2) those ESTs and cDNA libraries that exist have not been extensively screened for gene-based SSRs with the exception of the work of Blair et al. [6] and Hanai et al. [7,8]. Yet, common bean is essential for micronutrient nutrition and is adaptable to marginal areas for small-scale farm agriculture despite problems of low phosphorus soils or other abiotic constraints [9,10] and a range of diseases and pests [11]. Therefore a more complete toolbox of molecular tools for this crop is needed especially in the case of gene-based markers which can be based on SSRs polymorphisms as will be discussed here.

In our efforts to accumulate a larger set of genic SSRs, we previously constructed a leaf based cDNA library from Andean genotype G19833 [12] and used a hybridization approach to discover SSRs of various di-nucleotide and tri-nucleotide motifs and develop microsatellites from this library in the BMc (Bean microsatellites from cDNAs) series [6]. We have also recently developed two additional root based cDNA libraries under high and low phosphorus conditions from the Mesoamerican genotype DOR364, the other parent of the mapping population of Blair et al. [2] and sequenced ESTs from the libraries to discover new SSRs.

The EST sequencing of these libraries is used in this research as the basis for determining the frequency of SSR sequences in root expressed genes as opposed to leaf expressed genes and for adding to the BMc series of microsatellites through an *in silico *approach to microsatellite discovery as described by Varshney et al. [13] for some species of cereals. EST-SSRs are more common in cereals than they are in legumes [14-16].

Apart from our efforts, currently there are approximately 70,000 other EST sequences from common bean including collections from Ramirez et al. [17], Melotto et al. [18] and Thibivilliers et al. [19] along with small groups of GenBank entries and a wish-list of further EST efforts [9]. However most of these libraries have not been screened nor compared for SSR markers. The Melotto et al. [18] libraries from anthracnose infected common bean leaves which contain together approximately 4,000 unigenes has been screened for microsatellites, yielding a set of 140 EST-based SSRs for Hanai et al. [7,8], although many of these have been used for genetic mapping rather than for germplasm characterization.

The objective of this research was to evaluate the frequency of microsatellites in sequences from different leaf and root EST libraries made in our laboratory, comparing the types of microsatellites from each source tissue. From there we developed the most promising microsatellite loci as gene-based SSR markers that we added to the BMc series of markers [6]. To validate these BMc markers we compared their ability to detect polymorphism in a standard germplasm panel of 18 mapping parent genotypes, which included Mesoamerican, Andean, wild and cultivated accessions that were useful for determining polymorphism information content of the different groups of markers. A final objective was to determine whether any difference in the ability to uncover polymorphism existed between the newly developed BMc markers found in the random EST sequencing versus BMc markers developed by our previous hybridization-based approach.

## Methods

### cDNA library and EST sequencing

Three cDNA libraries were searched for microsatellite containing sequences. These libraries were based on 1) mRNA from leaf and stem tissues as described in Blair et al. [12] and Ramirez et al. [17] for the genotype G19833; 2) a library that was made in the pBS-SKII vector from mRNAs of hydroponically grown DOR364 roots which were produced under low phosphorus (LP) conditions and 3) a final library also made in the pBS-SKII vector from mRNAs of hydroponically grown DOR364 roots but which were from a high phosphorus (HP) treatment.

In total, 1308 ESTs were sequenced from the G19833 leaf and stem tissue library: 540 sequences (GenBank entry BQ481427-BQ481965) from Blair et al. [12] and 768 sequences (HS089176-HS089943) sequenced for this study. Meanwhile, a total of 1815 ESTs were sequenced from the DOR364 root tissue libraries: these being 862 from the HP library (GenBank entries, HS103978-HS104836) and 953 from the LP library (GenBank entries, HS103028-HS103977). Clones from all cDNA libraries were sequenced from the 5'end using BigDye chemistry (Applied Biosystems by Life Technologies; Carlsbad, CA) and di-deoxy-based Sanger sequencing reactions at the Clemson University Genomics Institute (CUGI). All EST sequences were screened for microsatellites to be assigned to the BMc series as described in Blair et al. [6] and with the methods given below.

### SSR identification, primer design and microsatellite amplification

SSRs were identified by screening the EST collections with SSR Locator [20] with the default option of 1 to 6 nucleotide repeats. Primers were designed using Primer3 [21] with the following conditions: optimum primer length of 20 nucleotides (nt, minimum 18 nt - maximum 26 nt), optimum melting temperature of 50°C (min. 45°C - max. 55°C), an optimum product size of 125 base-pairs (bp, min. 100 bp - max. 350 bp) and an optimum G/C content of 50% (min. 45%- max. 55%). New markers were submitted as STS entries to GenBank and are listed in the Additional file 1 (Table S1).

PCR reaction conditions for all newly designed BMc markers and for the 248 BMc markers from Blair et al. [6] are as follows: 30 ng of genomic DNA, 0.16 μM of mixed forward and reverse primers, 1X Buffer (10 mM de Tris-HCl pH 8.2, 50 mM KCl, Triton 0.1%, BSA 1mg/ml), 1.5 mM MgCl2, 0.2 mM dNTPs and 1 U *Taq *polymerase in 12 μL reaction volumes. Amplification conditions were based on those described in Blair et al. [6,22] with 35 cycles and 47°C annealing temperature. PCR reaction products were run on PTC-200 thermal cyclers (MJR, Bio-Rad Laboratories; Hercules, CA) and then denatured at 94°C and run on 4% polyacrylamide gels (5M urea, 0.5X TBE) in metal backed Owl T-Rex vertical S3S gel units (Thermo Fisher Scientific Inc; Waltham, MA) at constant 120 W. Silver staining was performed as described in Blair et al. [22,23].

### Germplasm survey

The set of genotypes used for the polymorphism survey in this study was based on a germplasm panel of 18 genotypes described in Blair et al. [22] as panel I. Both the DOR364 genotype, a Mesoamerican gene pool advanced line from the International Center for Tropical Agriculture (CIAT), and the G19833 genotype, an Andean gene pool Peruvian landrace in the FAO collection at CIAT were obtained from the gene bank in the Genetic Resources Unit (GRU), and used in a polymorphism survey since these were the sources of the EST libraries we screened for microsatellite loci. Along with these two genotypes the germplasm survey included nine more domesticated Mesoamerican accessions and varieties (G3513, G4825, G11360, G11350, G14519, G21212, BAT477, BAT881 and DOR390), four other domesticated Andean accessions or varieties (G21078, G21657, G21242, Radical Cerinza) and three wild accessions (G19892, G24390 and G24404) representing Andean, Mesoamerican and Colombian wild sub-populations) which were also provided by the GRU.

DNA extraction consisted in a CTAB based mini-prep procedure as described in Afanador et al. [24] using bulk leaf tissue from four greenhouse grown plants per genotype or line. Since the accessions were from lines separated by seed color and maintained at the gene bank, or from advanced lines from the CIAT collection, we assumed homozygosity for all the germplasm but noted any double banding that could indicate a heterozygote or heterogeneous mixture from the four plants. Although beans are a highly inbreeding species (95 to 99%) some outcrossing occurs occasionally so there can be some within accession or intra-population variation and this would be observable in any lanes containing more than one band, representing more than one allele in seeds of the accession.

### Data analysis

Allele sizes were estimated for the survey panel and mapping gels based on comparison with 10 and 25 bp molecular weight ladders that were distributed twice on each silver stained gel. A neighbor-joining (NJ) dendogram was constructed with the proportion of shared alleles coefficient and matrix of alleles and genotypes for the survey panel with the software programs Darwin [25]. Polymorphism information content (PIC) was calculated for each marker with Powermarker [26].

## Results

### Comparisons of EST-SSR repeat types and marker development

Among the SSR motifs identified (Table 1), tri-nucleotides were the most common with 99 out of 184 found (53.8%) while di-nucleotide repeats were the second most common with 57 out of 184 found (30.9%). Meanwhile, only a few tetra-nucleotide (23) and penta-or hexa-nucleotide (5) SSRs were observed. Across all the EST sequencing sets the percentage of ESTs containing SSRs varied from 3.5 to 11.9% with the highest number found in the first sequencing of the leaf library and the least in the second sequencing of the leaf library which may have been due to sampling differences. The numbers of SSRs per ESTs in the two root libraries were similar, with 5.4% for the HP library and 4.8% for the LP library. When comparing the leaf versus root tissues we found that 6.9% of the leaf ESTs had SSRs while 5.1% of the root ESTs had SSRs so the values were similar overall. More tetra-nucleotide SSRs were found in leaf ESTs than in root ESTs while the number of di-nucleotide SSRs in relationship to the number of ESTs sequenced was similar in the two EST collections. Similar numbers of tri-nucleotides were found in ESTs from each type of tissue.

**Table 1:** Microsatellites, simples sequence repeat (SSR) class and motif type found with in EST collections positive for SSR loci

Tissue/Library type	Genotype/Gene pool	EST collection/author	**EST No**.	EST-SSRs found	2-nt	3-nt	4-nt	5/6nt	% EST-SSRs	GenBank entries for ESTs
										

Leaf cDNA	G19833	Blair (2002)	540	64	9	34	21	0	11.9	BQ481427-BQ481965,
Leaf cDNA	G19833	Blair (this study)	768	27	10	16	0	1	3.5	HS089176-HS089943
**subtotal**	**Andean**	**NA**	**1308**	**91**	**19**	**50**	**21**	**1**	**7.0**	**NA**

										

HP root cDNA	DOR364	Blair (this study)	862	47	20	23	2	2	5.5	HS103978-HS104836
LP root cDNA	DOR364	Blair (this study)	953	46	10	26	0	2	4.8	HS103028-HS103977
**subtotal**	**Mesoamerican**	**NA**	**1815**	**93**	**30**	**49**	**2**	**4**	**5.1**	**NA**

										

**grand total**	**Andean/Meso american**	**NA**	**3123**	**184**	**57**	**99**	**23**	**5**	**5.9**	**NA**

When comparing the specific motifs for SSRs found in each set of ESTs (Table 2) we observed similar frequencies of specific types of motifs among the di-nucleotides but different frequencies of specific types of motifs among the tri-nucleotides. Overall among the di-nucleotides AG/CT/GA/TC microsatellites were much more common than other types of di-nucleotide motifs with 41 out of 57 of these SSRs (71.9%). The next most common was the AT/TA microsatellites with 12 out of 57 of these SSRs (21.1%) while no CG/GC microsatellites were found. Only four AC/GT/CA/TG microsatellites were found constituting only 7.0% of the total di-nucleotide repeat motif SSRs identified. Among the tri-nucleotide SSRs, AAG/AGA/GAA/TTC/TCT/CTT was the most common motif with 23% of the total followed by AGG/GAG/GGA/TCC/CTC/CCT with 16%. The CGC and ATA-rich microsatellites were the least common with all others being intermediate.

**Table 2:** Percentage of SSR types across four EST collections

SSR Type/Genotype/Tissue source	G19833 set 1leaf cDNAs	G19833 set 2 leaf cDNAs	DOR364 root HP	DOR364 root LP	**Total SSR and Seq**.
*Di-nucleotide motifs^1^*					
ac/gt/ca/tg	11.1	10.0	0.0	11.1	7.0
ag/ct/ga/tc	88.9	50.0	85.0	61.1	71.9
at/ta	0.0	40.0	15.0	27.8	21.1
gc/cg	0.0	0.0	0.0	0.0	0.0

*Tri-nucleotide motifs*					
aag/aga/gaa/ttc/tct/ctt	11.8	25.0	30.4	30.8	23.2
aat/ata/taa/tta/tat/att	2.9	0.0	4.3	7.7	4.0
aac/aca/caa/ttg/tgt/gtt	8.8	6.3	13.0	3.8	8.1
acc/cac/cca/tgg/gtg/ggt	17.6	6.3	4.3	19.2	13.1
agc/cag/gca/tcg/gtc/cgt	17.6	12.5	13.0	3.8	12.1
agg/gag/gga/tcc/ctc/cct	20.6	6.3	17.4	15.4	16.2
atc/cat/tca/tag/gta/agt	2.9	37.5	8.7	11.5	12.1
ccg/gcc/cgc/ggc/cgg/cgc	2.9	0.0	4.3	0.0	2.0
gac/cga/acg/ctg/gct/tgc	14.7	6.3	4.3	7.7	9.1

Motifs are distinguished for di- and tri-nucleotide based simple sequence repeats (SSRs) used to create new BMc markers.

^1^Complementary sequences for a given motif are given and were the basis for grouping of di-nucleotide and tri-nucleotide motif SSR.

In the effort to develop additional cDNA-derived microsatellites, we added 120 new BMc (bean microsatellites from cDNAs series) markers to the 248 previously developed BMc markers [6]. Among the microsatellites, the first seventeen (BMc1 to BMc17) were developed from leaf cDNAs in the library described in [6,12] and as shown in the Additional file 1 (Table S1). A second set of leaf cDNA derived microsatellites from our second EST sequencing effort in this library were designated as BMc18 to BMc27. Meanwhile, 47 microsatellite markers (BMc28 to BMc74 plus BMc77 to BMc109 except BMc55 and BMc59) were developed from the HP root library and 46 other microsatellite markers (BMc55, BMc59, BMc75, BMc76 and BMc78 to BMc108 as well as BMc110 to BMc120) were developed from the LP root cDNA libraries. In summary the largest number of new cDNA derived microsatellites were found in the root libraries (93 out of the 120) compared to the leaf library (27 out of the 120).

Among the newly developed markers 50 were based on di-nucleotide repeats, 66 on tri-nucleotide repeats and 4 on tetra-, penta- or hexa-nucleotide repeats which we generally avoided for primer design (Table 3). The new markers produced expected product sizes from as small as 80 to as large as 298, although the majority were designed to be small PCR amplicons to avoid the possibility of including exons. The average number of repeats in the BMc markers (including both compound and simple SSRs) was 6.8 repeats per microsatellite but this varied from an average of 9.1 for di-nucleotide motifs to 5.3 for tri-nucleotide motifs and 4.3 repeats for other tetra, penta or hexa-repeat based motifs.

**Table 3:** Summary of the motif and polymorphism characteristics of microsatellites found in BMc markers

BMc marker types	Leaf EST source	Root EST source	Number of SSRs	Percentage of total
Di-nucleotide based	10	40	50	41.7
Tri-nucleotide based	16	50	66	55.0
Tetra, penta or hexa-nt based	1	3	4	3.3
Multi-copy	0	6	6	5.0
Non-Amplifying	6	10	16	13.3
Monomorphic in survey	12	47	59	49.2
Polymorphic in survey	9	30	39	32.5
Polymorphic in cultivated	7	27	34	28.3
Polymorphic in wild only	1	4	5	4.2

BMc markers developed from leaf and root expressed sequence tags (EST) are separately and jointly considered for number of simple sequences repeats (SSRs) and percentage of total.

**Figure 1:**
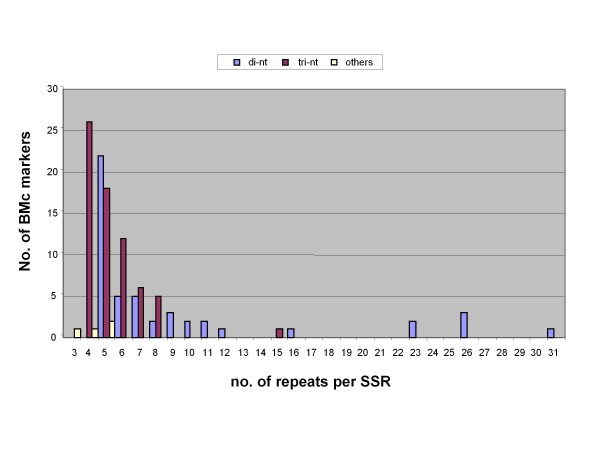
**Distribution of repeat sizes for BMc markers**. Bars of different colors show the number of BMc markers from di-nucleotide, tri-nucleotide and other (tetra-, penta- and hexa-nucleotide) categories with different numbers of repeats.

The highest repeat numbers were found for BMc70 (31 repeats) and BMc58 (26 repeats) as well as BMc30 and BMc33 (23 repeats, each); all of which were based on di-nucleotide motifs; the first and last two based on GA_n _with the second based on CA_n_. Surprisingly, there were few long AT_n _microsatellites, with the exception of BMc3 (26 repeats), but this may be due to the genic nature of the microsatellites developed. The distribution of repeat sizes among the BMc markers was skewed generally to the smaller number of repeats; the reader is reminded that the minimum number of repeats for di-nucleotides was five and for tri-nucleotides was four while for all other types it was three (Figure 1). Interestingly, a small group of di-nucleotide microsatellites with large numbers of repeats were found to the right of the graph and greater skewing of di-nucleotide compared to tri-nucleotide microsatellites was found towards the left of the graph.

**Figure 2:**
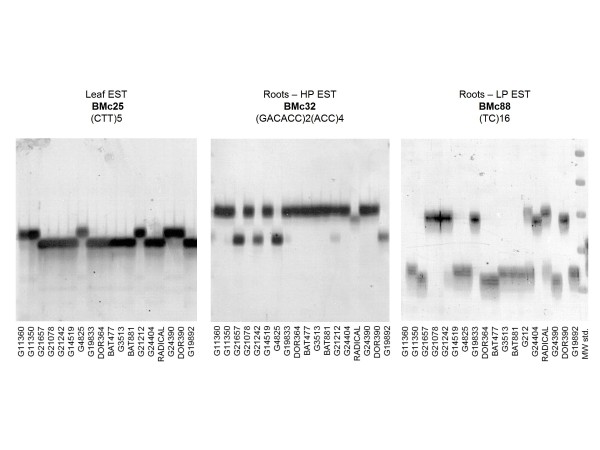
**Examples of germplasm survey for 18 genotypes evaluated with leaf and root EST library derived BMc markers**. Markers for both low phosphorus (LP) and high phosphorus (HP) expressed root genes are shown as well as the names of the genotypes used in the germplasm survey. Example of a molecular weight standard of 10 base pair (bp) differences is shown to the far right.

When comparing the source tissue for the BMc markers, the ratio of di-nucleotide and tri-nucleotide markers was similar for root and leaf derived microsatellites (Table 3). These ratios held true for the proportion of markers that had problems of non-amplification (16 out of 120) or that were multi-copy (6 out of 120). The markers showing multiple monomorphic banding were BMc30, BMc58, BMc60, BMc70, BMc92, and BMc96. The ratio of simple to compound SSRs was 102 to 18 among the new BMc markers, 85% and 15% of the total number of markers, respectively. Among the compound repeats many were just due to an interruption of the same repeat (7 out of 18). Therefore the percentage of truly compound repeats was even lower (11 out of 120) corresponding to 9.2% and the vast majority were simple, perfect motif SSRs. Amplification strength was similar for SSRs of different motifs and repeat lengths (Figure 2).

### Genetic diversity detected

As described above, out of the 120 new BMc markers a total of 98 microsatellites amplified well in the survey panel and these were used for polymorphism survey for the germplasm panel and diversity analysis. In this final set of 98 functional markers, 59 (60.2%) were monomorphic and 39 (39.8%) were polymorphic. The average PIC value of the new polymorphic BMc markers was 0.310 and ranged from 0.099 for the least polymorphic markers to 0.657 for the most polymorphic marker (BMc70).

Polymorphism comparison of the di-nucleotide and tri-nucleotide markers showed that they had similar average PIC values (0.131 and 0.125, respectively) when considering both monomorphic and polymorphic microsatellites together. A similar situation was observed when considering only polymorphic microsatellites, where di- and tri-nucleotide based markers again had similar PIC values (0.322 and 0.301, respectively). None of the tetra-, penta- or hexa-nucleotide repeat-based markers was polymorphic.

Polymorphic markers were in similar proportion (38% in each case) for the BMc markers from leaf ESTs (8 out of 21 functioning markers) and for the BMc markers from root ESTs (30 out of 77 functioning markers). Interestingly some polymorphic root-derived BMc markers (BMc30, BMc40, BMc58, BMc60 and BMc70) showed monomorphic background bands suggesting they were members of gene families with different degrees of diversity in different homologs.

A set of five microsatellites (BMc17, BMc36, BMc44, BMc61 and BMc68) was only polymorphic in the wild accessions but not in the cultivated accessions or varieties. These markers had relatively low PIC values of 0.099 to 0.157. From the 368 current BMc markers, including the 248 from the previous study of Blair et al. [6] and the 120 described here, a total of 209 (56.8%) of the BMc markers yielded monomorphic results while 159 (43.2%) produced polymorphic results in the germplasm survey.

**Figure 3:**
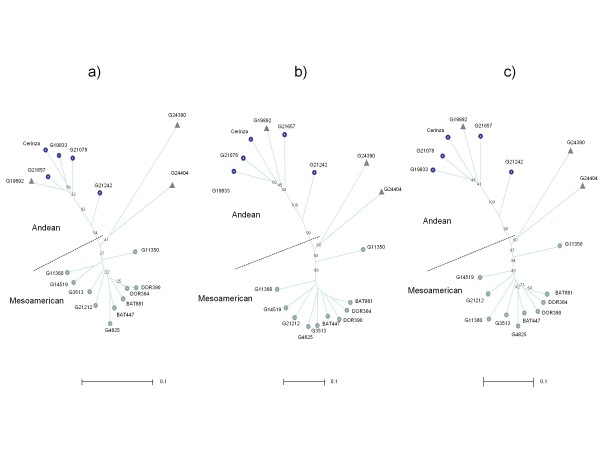
**Neighbor joining dendrogram of relationships between Andean, Mesoamerican, cultivated and wild accessions of common bean**. Dendograms are based on different groups of cDNA derived markers: a) newly developed BMc markers 1-120; b) previously developed BMc markers 121-368 from Blair et al. (2009a) and c) all BMc markers from 1-368. The Andean and Mesoamerican genepools are indicated in each case with a subdividing dark line that separates the dendograms in two and with different shades of circles at the end of the branches for cultivated accesssions. Wild genotype accessions are indicated with triangles at the end of the branches and included G19892 (from Argentina), G24390 (Mexico) and G24404 (Colombia).

The average PIC value of the full set 368 BMc microsatellites was calculated to be 0.291, while for all those that were polymorphic the PIC value was 0.424. When the diversity analysis with the newly-developed, cDNA-derived markers BMc1 to BMc120 was undertaken (Figure 3a) we found that the Andean and Mesoamerican gene pools represented the main axis of the neighbor joining tree upon which two of the wild accessions then showed a divergence from the cultivated genotypes. The Argentinean accession G19892 grouped within the Andean genepool, while the highly diverse Colombian (G24404) and Mexican (G24390) accessions were near the division of the two gene pools. These results agree with the neighbor joining analysis of Blair et al. [6] who evaluated the markers BMc121 to BMc368 (Figure 3b).

When the results of the phylogeny analysis of the newly developed makers were combined with the previous markers from Blair et al. [6] an even clearer picture of the associations emerged (Figure 3c). Although all dendrograms showed very highly supported nodes for the separation of the two main gene pools and the two wild accessions; in the combined analysis, we found very high bootstrap values (ranging from 90 to 100%) based on the strength of the total set of markers evaluated.

## Discussion

The major achievements of this research were 1) to evaluate microsatellite frequency in three cDNA libraries from root and leaf tissues with one of the root libraries developed for the abiotic stress of low phosphorus and 2) to create additional genic microsatellite markers based on low-level sequencing of these EST libraries to use in a polymorphism survey both to understand common bean genetic diversity and to understand the differences in various microsatellite types from different sources and their ability to uncover bean diversity. The creation of new genic microsatellites is especially pressing as only about 230 [2,7,8,27,28] had been reported before we started our work on the design of BMc microsatellite markers.

In total we have now designed 368 genic microsatellites in the BMc series between the efforts of this study and the previous work of Blair et al. [6]; all BMc markers were designed from cDNA libraries made from different tissues of the mapping population parents used by Blair et al. [12,23]. In addition, with this study we have created BMc markers from two different genotypes including G19833 and DOR364 and from leaf tissue and root tissues subjected to low or high phosphorus conditions. The advantage of having markers developed from sequences of both genotypes resides is the fact that the Andean G19833 is being used for whole-genome shotgun sequencing and the Mesoamerican DOR364 provides a commercially useful tropical, small red seeded counterpart to the Andean genotype and to black beans which have been better studied in terms of agronomy as well as EST development [17].

In addition, both marker types from both genotype sources are useful for evaluation in the reference map based on DOR364 × G19833 studied by Blair et al. [2,23] which is linked both the UC-Davis [29] and Univ. of Florida [30] genetic maps. In terms of the practical use of the microsatellites, the PCR amplification strength was similar for SSRs of different motifs and repeat lengths, which may be typical of gene-derived microsatellites and distinct from genomic microsatellites as first suggested by Blair et al. [22].

In our previous study of cDNA derived microsatellites [6] we found that uniformly strong PCR products were obtained with the specific primer sets around the SSR loci in cDNA sequences. In comparison, amplification with non-gene based microsatellites is prone to some pitfalls as discussed by Blair et al. [23] for AT-rich microsatellites and Blair et al. [31] for hybridization derived genomic microsatellites. Differences between genic and different kinds of genomic microsatellites have been observed for other marker sets as well [7,32,33]. Although the SSR and EST sequencing effort from most of these projects has been small it is useful to have added their sequences to GenBank to compare in the future to larger EST collections from Ramirez et al. [17], Melotto et al. [18] and Thibivilliers et al. [19] as well as future genomic sequences for common bean or related species. Furthermore the possible role of microsatellites as promoters or gene expression enhancers especially in root genes where many AG_n _microsatellites were found could be studied.

In terms of other di-nucleotide motifs, the lack of GC microsatellites has been observed before within the bean genome [6,31], while AT-rich microsatellites were not expected to be found in genic sequences neither as di-nucleotides nor tri-nucleotides such as those studied by Blair et al. [23]. There were only a few AC_n _based microsatellites which was surprising given that enrichment for this motif has yielded about the same number of markers as enrichment with AG_n _or GA_n _based probes [7,34]. Among the tri-nucleotide motifs it appears that AAG (23), ACC (12), AGC (12), AGG (16) and ATC (12) microsatellites are the most common and this may have to do with their frequency in triplet codon use for amino acid incorporation into polypeptides. Additionally, open reading frames are known to have a higher GC percentage than non-translated regions [35] which might favor tri-nucleotide motifs such as ACC, AGC and AGG. Compared to the results of Blair et al. [6,31] the ratio of tri-nucleotide to di-nucleotide motifs was fairly high (99 *versus *57 in total). Perhaps this was due to a majority being located in the open reading frame rather than in untranslated regions of the original mRNA transcripts represented by the cDNA sequences.

In the second step of this study, we analyzed the potential of two different groups of BMc markers, one from cDNA clone sequencing (120 BMc markers) and one from cDNA hybridization with SSR motifs (248 BMc markers developed from 497 positive cDNA clones) to be used in phylogeny analysis. The full group of markers, therefore, included a total of 368 BMc microsatellites all evaluated against the same germplasm survey from Blair et al. [6]. In that evaluation, genetic diversity was reliably predicted by both types of cDNA based BMc microsatellites. Both sets of markers were useful in separating the Andean and Mesoamerican genepool and accurately placing the wild accessions within each genepool. Two wild accessions (Colombian and Mexican) were separated from the cultivated accessions. Similar results were found with the same diversity panel in Blair et al. [6].

In summary, cDNA derived markers seem to be very useful for diversity analysis due to the fact that they are derived from genic sequences that are conserved and are highly transferable between different accessions of beans. They were critical in recent studies of diversity in both dry and snap bean cultivars of *Phaseolus vulgaris *[36,37]. Therefore, in the future we plan to analyze the frequency of gene-based microsatellites in larger collections of ESTs such as those of Ramirez et al. [17] or Thibivilliers et al. [19] which surpass the numbers of ESTs evaluated in the libraries we used here. It will be interesting to see if SSR frequency is similar or different for the multiple libraries used by the first of these authors or the larger set of ESTs from a single rust-infected leaf library evaluated by the second research group. One lesson from this microsatellite evaluation is that it is important to test new markers for consistent patterns of genetic diversity detection. We also plan to test the gene-derived markers in related *Phaseolus *species.

## Conclusions

In terms of the evaluation of genetic diversity we found that genic microsatellites from both EST sequencing and hybridization based approaches performed equally well in distinguishing Andean and Mesoamerican genepools and the Argentinean, Colombian and Mexican wild beans as separate accessions. Therefore, these markers can be used for diversity analysis and for breeding especially in crosses between wild and cultivated beans or between genepools. We expect that next generation sequencing will make the discovery of new transcriptome-based SSRs even easier than the two approaches used so far. Nonetheless, the utility of cDNA derived microsatellites for diversity analysis is well established and is perhaps best explained due to their conservation and slower rate of evolution than genomic microsatellites. In summary, gene-based or 'genic' microsatellites appear to be especially useful for genetic analysis of common bean and it would be ideal to have a larger set of these markers for functional diversity analysis and perhaps association mapping once they are genetically mapped which will be the subject of a separate manuscript to define the regions of the genome that are part of the transcriptome. Finally, these gene-based markers may be the keys to selection of specific traits as they represent expressed genes some of which are likely to have multiple functional alleles with diverse phenotypes as a result. Simple sequence repeats in promoter regions have sometimes been found to be important in controlling gene expression and this may be the case for some of the genic markers discovered in this study as well.

## Authors' contributions

MWB conceived and organized the study and wrote the manuscript. NH and MCC and MCG performed the laboratory work for BMc marker evaluation. NH and MCC helped in writing the manuscript and preparing tables and figures. MCMT contributed to writing and designed the primers. FP and MCMT constructed, arrayed and screened all the libraries at CIAT and CUGI. JT and RW assisted with library preparations at CUGI. All authors read and approved of the manuscript.

## Supplementary Material

Additional file 1 **Supplementary Table S1. Primer sequences and simple sequence repeat motif for new set of cDNA-derived BMc (Bean micorsatellite derived from cDNA sequence) series markers**. GenBank entry, predicted product size based on EST sequence and polymorphism information content (PIC) given for each marker. Click here for file
