# Supplementary material for: Gene-based SSR markers for common bean (Phaseolus vulgaris L.) derived from root and leaf tissue ESTs: an integration of the BMc series
Source: BMC Plant Biol. 2011 Mar 22;11:50. doi: 10.1186/1471-2229-11-50 (PMC3068092; doi:10.1186/1471-2229-11-50)
Supplement: Additional file 1 — Supplementary Table S1. Primer sequences and simple sequence repeat motif for new set of cDNA-derived BMc (Bean micorsatellite derived from cDNA sequence) series markers. GenBank entry, predicted product size based on EST sequence and polymorphism information content (PIC) given for each marker. [file 1471-2229-11-50-S1.DOC]

Supplementary Table 1. Primer sequences and simple sequence repeat motif for new set of cDNA-derived BMc (Bean micorsatellite derived from cDNA sequence) series markers. GenBank entry, predicted product size based on EST sequence and polymorphism information content (PIC) given for each marker.

| **Primer Name** | **GenBank** | **Left Primer** | **Right primer** | **Motif** | **Product Size** | **PIC^1^** |
| --- | --- | --- | --- | --- | --- | --- |
| BMc1 | HO212318 | TAGGGGGTAGAGAGGGAGGA | CCAAGCTCCCACCCTAATAA | (AG)9 | 125 | 0.000 |
| BMc2 | HO212319 | TCTGACTCTGGGACCCTCAC | CCTTCTTCTTCTCTGGGGTTG | (CAT)8 | 135 | 0.647 |
| BMc3 | HO212320 | TTGTGGTATATCTTAATCGCCAAG | AGCATCCGGATTAAGTGCAG | (TA)26 | 147 | 0.099 |
| BMc4 | HO212321 | GGAGTAGGACGGTGTGGTATGT | CTAGCTCCATCTCCCATCCTT | (AG)10 | 97 | 0.000 |
| BMc5 | HO212322 | TGCTAGGGGAGGAGCTAACA | GCCTTCTCCATGGTGGAAAT | (ATC)7 | 142 | 0.352 |
| BMc6 | HO212323 | TTGGCTTTGGTGGATACTCA | CAAAACCAAGAAACCCATCAA | (ATG)5 | 139 | 0.099 |
| BMc7 | HO212324 | GATGATGAGGATGGAGTTGAG | CCCTCCGTACTCGTTAACCTC | (ATG)6 | 128 | 0.248 |
| BMc8 | HO212325 | GCCATGCTTAGAGGTCGTGT | TTTCCGACATGCATAAACACA | (AT)5 | 111 | 0.195 |
| BMc9 | HO212326 | CCTTCTGCTCTACCCATTTGA | CCAAGAGAGTGATCGTTGGT | (TC)6(CT)2 | 119 | 0.352 |
| BMc10 | HO212327 | GCGCCCACGCTTATCAAT | CAATAGCCGTTGGAAGCAGT | (CT)7 | 145 | na |
| BMc11 | HO212328 | CCGTCGATTCCCATAAAGTG | CGCACTGAGAAGAGGGAGAA | (CA)5 | 136 | 0.000 |
| BMc12 | HO212329 | TTTCCTATGCGCCCAGCG | GGAATCGGAATATCGGGAAG | (CCA)5 | 98 | na |
| BMc13 | HO212330 | GCGTCCGTTGGCTTGT | AAGCTTTTGCCCTGTCCAA | (CTTCT)5 | 150 | na |
| BMc14 | HO212331 | TGTCTGCACAGCTCTGATTC | CCTTTCCAACACACCACTGA | (AT)7(GA)4 | 127 | 0.000 |
| BMc15 | HO212332 | AAGAGGCCGAGAAGAAGAGG | GCTTCCGTCTTTGATTCACC | (TGA)7 | 127 | na |
| BMc16 | HO212333 | CACAGAGCAACCTTGTTCCA | GGCCAAGTGGAGGTTGTTAT | (TAC)5 | 97 | 0.000 |
| BMc17 | HO212334 | TGCAGGGTTCACAGTCAGAG | CAAACCAGCAGCAACAAGAG | (ACA)2(CAA)5 | 103 | 0.099 |
| BMc18 | HO212335 | CCGGCTTCTTTCTCAAACTC | GAGGGTGATTGCAGGAGACT | (TCT)4 | 140 | 0.000 |
| BMc19 | HO212336 | CTCTGTCCTCTCCATGCTCA | CAGTGGAGACGCTGATCG | (TCC)5 | 141 | 0.000 |
| BMc20 | HO212337 | GTGGGAGCAGAACAGCAAGT | ACGGTTAACGGTCGTCACAG | (AAG)5 | 97 | 0.000 |
| BMc21 | HO212338 | ATCATGCCTGTCCAGAAACA | GACTTCCTTGCTGCACTTGA | (TCG)4 | 117 | 0.000 |
| BMc22 | HO212339 | CACGCGTCCGTTCTCTTC | TGATGCCTTTGACTTCACCA | (AGC)4 | 179 | na |
| BMc23 | HO212340 | TGTGGTATATCTTAATCGCCAAG | CACCGTAAGCAAATTCAGCA | (AT)26 | 162 | 0.447 |
| BMc24 | HO212341 | GAAACTTCAGAAGCAGCAGCA | CTGGCTTCCAGATTATTATGACG | (GAG)4 | 162 | na |
| BMc25 | HO212342 | GATGGCTTCCATGGCTTCTA | GCTGTGATGCAGAGAAAGAGAG | (CTT)5 | 116 | 0.346 |
| BMc26 | HO212343 | TGTTGACTTGGGCTATGAGGT | GCCACGAGCAAGAAGATCAG | (AG)5 | 130 | 0.000 |
| BMc27 | HO212344 | TAGAGCACCACTCTCGCACT | AGTCCAGACGCCAGAAACAG | (TTC)4 | 124 | 0.000 |
| BMc28 | HO212345 | AATCCTCACGTTGCGTCATT | ACCTCTTTCTCCTTCCCTGAG | (TCAT)2(AT)3 | 120 | 0.000 |
| BMc29 | HO212346 | AATGGACCACGCTGCTTTAT | TTGGTACATGCACTAAGCTGAA | (CT)5 | 80 | 0.000 |
| BMc30 | HO212347 | GAGAGAGAGAGAGAGAGAGAGTAGAG | GGGCTGAGAGGGCTATGA | (AG)23 | 119 | na |
| BMc31 | HO212348 | GCCTGTGCCTCTCCAAAGT | AAAGTGCCAGAAGCTGTTCC | (ATT)4 | 168 | 0.000 |
| BMc32 | HO212349 | TCCTCTTCTTCAGCCCAATG | TTCCCAGCTCTTCCCAGA | (GACACC)2(ACC)4 | 140 | 0.384 |
| BMc33 | HO212350 | GGCAATCAATTGACACAGACA | GTTGGAGCAGCAGCATTTC | (CT)23 | 165 | 0.426 |
| BMc34 | HO212351 | TGCAGCTTCTCGTTTCATCA | CGCCATAACAACAATGAAGG | (CTG)6 | 119 | 0.473 |
| BMc35 | HO212352 | TTCTGCAGATGGATTTGACG | GGAAGGGCTTCCTCTGTGAT | (GA)5 | 114 | 0.000 |
| BMc36 | HO212353 | CACTGCCCATTACACTATGAGG | GGAAGGTCCAGAGTTGTTGG | (AT)5 | 128 | 0.178 |
| BMc37 | HO212354 | CCAGTGAGTCGGTTGTCATTT | TCCTCCAGAACTTGGAAACC | (TGT)4 | 138 | na |
| BMc38 | HO212355 | TGCATAGTATAAGAATTCCATTGC | TGAATTTCGGTGTGATCCAA | (TA)7 | 118 | 0.000 |
| BMc39 | HO212356 | TATGCTGGGAAAGGAGCATC | CCCTTGGTGTTTCCACCTC | (TGG)5 | 122 | 0.000 |
| BMc40 | HO212357 | AGAGAGCGGGAGACCAAGAG | GCACTCATTTGTTTGGTGGA | (GAG)5 | 164 | na |
| BMc41 | HO212358 | CACAGCCATAGCCAGAAACA | GCTTGGAAGCTGCAATTCTC | (AGC)4 | 121 | 0.000 |
| BMc42 | HO212359 | GGGCTGTTGTATTAGGCTTGG | TGGAGGGTTGAAAGTGGAAC | (CAA)4 | 118 | na |
| BMc43 | HO212360 | TTCATCACTCTTCTCCAAATGC | GCTCAGAGGCTATTGTTCTAGGTC | (TTC)5 | 120 | 0.000 |
| BMc44 | HO212361 | GTAGTGTGGTGGAGGTGGTG | CCCAGTTTACAAATACAAATCC | (AGG)4 | 91 | 0.099 |
| BMc45 | HO212362 | GAAAGAATGTTAATGGTATTTCATACA | TTTCAGTGATATCCAATCAGATCA | (TA)5 | 84 | 0.000 |
| BMc46 | HO212363 | AGAGATGGATGCGCAGTGTT | TAGGGAGATACCGCAACTCG | (AG)5 | 114 | 0.000 |
| BMc47 | HO212364 | GGTGGAGGTGATGCGTAAAT | CCCTCCTTACACTCCTCATCC | (GGT)4 | 122 | 0.000 |
| BMc48 | HO212365 | CTTCTCTTCCTCTGCCTTCG | GGTACAACAACCGAAGTTCCA | (TC)5 | 87 | 0.000 |
| BMc49 | HO212366 | TTGCTCAACAGCAAACCAAC | GCGAAGGAATCTAAGACACGA | (CT)6 | 81 | 0.000 |
| BMc50 | HO212367 | ACGTTTCAGGTGGAAAGTGG | GGCTCGTGAATCTCCACAAT | (AGA)4 | 107 | 0.000 |
| BMc51 | HO212368 | GCAGCAGCAGAAACAACTACA | CGCTTCCTCTGTCTCCTTTG | (AG)5 | 105 | 0.000 |
| BMc52 | HO212369 | CAAGGGAGAGAGTGCTTTCG | TGTCCAACATCCAACCAAGT | (TG)5 | 94 | 0.000 |
| BMc53 | HO212370 | CACATGTGTCTCATACCCAACA | GAAGGGATAACCAAAAGGC | (ATG)4 | 103 | 0.000 |
| BMc54 | HO212371 | GTCCTTTGGCTGGACTATGC | GAGTTGGACAAGTAGGGAACTCA | (GAA)4 | 103 | 0.000 |
| BMc55 | HO212392 | ATTACAAGTCACCACCACCA | GGAGGGCTCTTGTAGTAGTAAGG | (CCA)3(CTA)3 | 112 | 0.000 |
| BMc56 | HO212372 | TGGGAAGAAAGAGACCCTGA | CCCTGCATCAGAAGTCCAA | (GA)5 | 118 | 0.000 |
| BMc57 | HO212373 | CCCCGCTACTACTACAAATCC | AGATCCATGTGGTGTGATGC | (CCT)6 | 82 | 0.000 |
| BMc58 | HO212374 | CCAGCAGGAGTTGAGCTTG | GAGGAAAGAGGGAAGAAGG | (TC)26 | 198 | na |
| BMc59 | HO212393 | GAAGGCCAAAGTTGCTGAAG | GCGAAGGAATCTAAGACACGA | (CT)6 | 122 | 0.000 |
| BMc60 | HO212375 | AGAGAACACACGCGCACAC | GGACTGCCATACCAGATGCT | (CT)5 | 128 | na |
| BMc61 | HO212376 | TCATCATTTCAAGGCCAACA | GCAGACCTGGACATCTCCTC | (AG)11 | 276 | 0.099 |
| BMc62 | HO212377 | GACCGTGTTTGGAAGCATTT | TCCATTTGCGTGGTTTAATG | (CT)5 | 94 | 0.000 |
| BMc63 | HO212378 | TTCCTTCTCCTCCTTCACCT | GGCTCCTCATAGTGGTCTTCA | (CTT)15 | 112 | 0.550 |
| BMc64 | HO212379 | GAATCAGACGCCTCGAGAAA | GCGGAAAGCTGGTCAGAGTA | (CTT)3(CTT)3 | 82 | 0.000 |
| BMc65 | HO212380 | GAACCCTCCATTGTCCAAGA | TCGTCATCTTCACCGTCGTA | (GAA)6 | 137 | 0.404 |
| BMc66 | HO212381 | GGGTTAGAGGCACAGCA | TGGAAGACGAGGGAACAG | (AG)5 | 127 | 0.239 |
| BMc67 | HO212382 | GCAACAATGGGTCAATTTCC | ACTGGAACCTGTTCAACAAA | (TAGCA)3(ATAG)2 | 158 | 0.000 |
| BMc68 | HO212383 | TTCAAAATTCCCTATTTACCTTCTTC | GCAGAACAACAATGCAGAGA | (TC)8(TTC)5 | 90 | 0.178 |
| BMc69 | HO212384 | CTCATACCAAAACTCAAACACAGT | AGAAGGTGGCCGTAGAAAGG | (GCA)5 | 127 | 0.178 |
| BMc70 | HO212385 | AGAGAGAGAGAGAGAGAGAGAGCAGA | CTGAATGATACGACGCAAGG | (GA)31 | 113 | na |
| BMc71 | HO212386 | ATCCGCTTGTTGGGTACAAT | GCAGGATAAACCTAAGGATCTTACAG | (TGA)4 | 80 | 0.000 |
| BMc72 | HO212387 | AAGAAGAAGAAAAGAAGAAGAAGAAG | AACCCATTTCCGACCAAT | (GAA)4 | 92 | 0.657 |
| BMc73 | HO212388 | CGTTGCCTTGTATCAGTTCC | CACGGAGTAGGGATTCTTGC | (CCG)4 | 109 | na |
| BMc74 | HO212389 | AGTCCCTTCACCACCTCCA | TGTGATGCAAGGGTGGTATC | (CCT)6 | 89 | 0.000 |
| BMc75 | HO212394 | CTATTTAGTGATTCCACACCTCACC | CAGGAGCACAAAATGTATCTTTCC | (TA)5 | 80 | 0.000 |
| BMc76 | HO212395 | AATTTGACGTGTGACGCTTTT | TTTCCATATAATGAGGTGTGTAAACT | (TCA)8 | 138 | 0.295 |
| BMc77 | HO212390 | ACGGTGAAGGTGCTGAGG | CCAACCAAACCACAGATTCA | (TTTC)4 | 298 | 0.000 |
| BMc78 | HO212396 | TCATCCTCATCGATGCAAAG | GAACTCTCAGCAACCGCATC | (TA)9 | 150 | 0.473 |
| BMc79 | HO212397 | CCATAAGAATTGAATGCGCTGA | TGTTTCAGGGTCAGGAAGGA | (TA)2(TTA)5 | 106 | na |
| BMc80 | HO212398 | GCAGTTGCTCTTCCTCAACC | AGGATTATCGGGTTCTCTGGA | (GAA)2(GAA)4 | 85 | na |
| BMc81 | HO212399 | TTGATCTTGAGGCTGCAGTG | GAGCAAACGTTGAGAAGCAA | (TC)5 | 106 | na |
| BMc82 | HO212400 | AACACGACCACCAGGGTATG | GGATAAGTGCAGCGAGGCTA | (CT)5 | 123 | na |
| BMc83 | HO212401 | GGCAGGAGGTTTGTGAATGT | TGTGGAGGAGGACTAGTTGC | (GCT)8 | 107 | 0.000 |
| BMc84 | HO212402 | AAGCTTTACCAAGCCTTCAT | CGGAAGCAAGTGAGGAGAAG | (CT)5(CT)1 | 129 | 0.000 |
| BMc85 | HO212403 | CCGAGGACCACCTCCA | GGCTTTCACTTCCACTTCCA | (CCT)6 | 130 | 0.000 |
| BMc86 | HO212404 | GGGCCCGTTTCACTTAGAA | CAAGCCACTTCATGCAGCTA | (AAC)4 | 100 | 0.000 |
| BMc87 | HO212405 | ATCTCCGGCAACCACTA | GGGTAGGGCTTGTGC | (CCA)4 | 170 | 0.000 |
| BMc88 | HO212406 | CACACCACAACACATCA | GTGTTCCTTCTCACAACA | (TC)16 | 123 | 0.624 |
| BMc89 | HO212407 | CGAATCTGCAGAGGCTGT | GAGACCTTCTTCGTGGAGGA | (CCA)5 | 130 | 0.000 |
| BMc90 | HO212408 | GAGTCAAGTTCGGTGAGAGC | AGTTCCAGAAGGGTTCAATAACA | (TA)10 | 167 | 0.621 |
| BMc91 | HO212409 | TCCACCATCACCTCACAAGA | AAGAAGCAGGAGTAGGAGAGGA | (TCC)4 | 110 | 0.504 |
| BMc92 | HO212410 | GGAAAAGAGATTTGGGAGGA | CCTGAGAATCACCACGAACA | (AGC)4 | 126 | na |
| BMc93 | HO212411 | AATCACAAGGCAAAGGTGCT | AGGGAACAAAACTTCAGACTTAGC | (TGG)5 | 99 | 0.000 |
| BMc94 | HO212412 | AGTGAGACGGCACAGAGAGA | GCACTATCACCGCCTTCTTC | (GA)5 | 120 | 0.346 |
| BMc95 | HO212413 | ACCTGCAACAACGCATGACT | GGTGTGGATTGTTGTTGTGC | (TTC)5 | 80 | 0.099 |
| BMc96 | HO212414 | CCGAGGACCACCTCCA | GGCTTTCACTTCCACTTCCA | (CCT)6 | 130 | na |
| BMc97 | HO212415 | CTCGCTTCTTCTGTACACTTCT | CATTCCACCAGCTCTTCCTG | (CTT)4 | 162 | 0.000 |
| BMc98 | HO212416 | AATTCCGATCTTCGATCTCT | TTCCAGCCGCCTGTATC | (CT)12 | 91 | na |
| BMc99 | HO212417 | ACGATTGGCATTCAAAGGAG | GGGTTAGGGTTAGGGTCAGG | (AAG)5 | 153 | 0.000 |
| BMc100 | HO212418 | TCACAGGATCTCGTTCTCTTTC | CAGTGGTGACGGGAGTTTCT | (CT)6 | 126 | 0.000 |
| BMc101 | HO212419 | TTTCATTCCCTGCTTTCTCC | TGAGGACCCGATCACACAC | (GAA)3(GAA)4 | 122 | 0.352 |
| BMc102 | HO212420 | ATCCTCCCATGGGTTCAAA | TCCCTTGTCTTGAAAGAACCTT | (AAT)4 | 127 | 0.000 |
| BMc103 | HO212421 | ACACACACACACACACACACA | GCACACACTTCATTACATTTCACAC | (TA)5(TA)2 | 191 | 0.141 |
| BMc104 | HO212422 | TCGTCACTACCTTGCACCTG | TCTCCCTATCACGACGCTTT | (AAG)7 | 104 | 0.000 |
| BMc105 | HO212423 | TTCGGGTCCTGGTTGTTTAG | CAATTTGTGCAACCCAAGGT | (GCT)2(CT)2(GCT)3 | 97 | na |
| BMc106 | HO212424 | GCGAAGGAGCTGCTCAATG | GCGTTTACGGAGGAATCTCA | (GTGGTG)3 | 114 | 0.000 |
| BMc107 | HO212425 | CCCAGTACCAGAACGAGCTT | CAGAGCTTCGCCATTTGAG | (GAA)5 | 126 | 0.099 |
| BMc108 | HO212426 | TTTGCTACTGTTGCTTTGTAATTT | TTTCTTTGAGGGAACCTTAGTT | (TCA)5 | 113 | 0.178 |
| BMc109 | HO212391 | TCCCTGTTCTCGCTCTCTTT | TTCCGTTGACTCCATTCTCC | (TC)9 | 139 | 0.000 |
| BMc110 | HO212427 | GGAGAAGGAGGAGAGATCCAA | ACACATTGCTCCCTGCATTT | (GAG)5 | 164 | 0.000 |
| BMc111 | HO212428 | GATTGCGTTGCCCATAACTC | TGCAGAATAGGCCTGAAGAAA | (CAT)4(ATGCA)2 | 141 | 0.000 |
| BMc112 | HO212429 | TGGAATAGGGAGCGGAGA | TTGATTCCATTCCGACATCA | (AC)3(AC)5 | 95 | 0.589 |
| BMc113 | HO212430 | GCAGAAATGTGACCTTCAACA | TCATCCGATCTAACGGTTCC | (CT)5 | 83 | 0.000 |
| BMc114 | HO212431 | TTAACCACCAACCTCATTTCC | TCCTCACATCAACACCAACAA | (TC)7 | 141 | 0.099 |
| BMc115 | HO212432 | CCTTGAAGCTGGAACCATGA | AAAGCAACCAGGTTTTGG | (AT)6 | 170 | 0.375 |
| BMc116 | HO212433 | GGAGATCGGAGTTCTCAACG | ATGGCGATAGAAAGGGAAGA | (TCT)4 | 119 | 0.346 |
| BMc117 | HO212434 | CGATTCTTTCGTTCGGAATC | CGCAGTCGTCCTCAATCATA | (CAT)4 | 167 | 0.099 |
| BMc118 | HO212435 | GCAGAAATGGGACCTTCAAC | CGTGTAGACGCAATCGTCAT | (CT)5 | 99 | 0.000 |
| BMc119 | HO212436 | CTCCATGCTGACCTCCATT | ATCAGAGTCGGGCACTTT | (CAC)5 | 134 | 0.105 |
| BMc120 | HO212437 | TGGTTTGGATCTGTGAAGCA | AATCTGGTGACGGCTTGTCT | (CA)7 | 98 | 0.000 |

^1^ Abbreviations: PIC = polymorphism information content, na = no amplification.
